# A Palindrome-Like Structure on 16p13.3 Is Associated with the Formation of Complex Structural Variations and *SRRM2* Haploinsufficiency

**DOI:** 10.1155/2023/6633248

**Published:** 2023-04-11

**Authors:** Alistair T. Pagnamenta, Jing Yu, Tracey A. Willis, Mona Hashim, Eleanor G. Seaby, Susan Walker, Jiaqi Xian, Emily W. Y. Cheng, Ana Lisa Taylor Tavares, Francesca Forzano, Helen Cox, Tabib Dabir, Angela F. Brady, Neeti Ghali, Santosh S. Atanur, Sarah Ennis, Diana Baralle, Jenny C. Taylor

**Affiliations:** ^1^Oxford NIHR Biomedical Research Centre, Wellcome Centre for Human Genetics, University of Oxford, Oxford, UK; ^2^Nuffield Department of Clinical Neurosciences, University of Oxford, Oxford, UK; ^3^Muscle team, Robert Jones and Agnes Hunt Orthopaedic NHS Trust Hospital, Oswestry, Shropshire, UK; ^4^Human Development and Health, Faculty of Medicine, University of Southampton, Southampton, UK; ^5^Genomics England, London, UK; ^6^Department of Metabolism, Digestion and Reproduction, Section of Genetics and Genomics, Imperial College London, London, UK; ^7^Department of Clinical Genetics, Guy's and St Thomas NHS Foundation Trust and King's College London, London, UK; ^8^West Midlands Clinical Genetics Service, Birmingham Women's and Children's Hospital, Birmingham, UK; ^9^Northern Ireland Regional Genetics Service, Belfast City Hospital, Belfast, UK; ^10^North West Thames Regional Genetics Service, London North West Healthcare University NHS Trust, Northwick Park Hospital, Harrow, UK

## Abstract

*SRRM2* encodes a splicing factor recently implicated in developmental disorders due to a statistical enrichment of de novo mutations. Using data from the 100,000 Genomes Project, four unrelated individuals with intellectual disability (ID) were identified, each harbouring de novo whole gene deletions of *SRRM2*. Deletions ranged between 248 and 482 kb in size and all distal breakpoints clustered within a complex 144 kb palindrome situated 75 kb upstream of *SRRM2.* Strikingly, three of the deletions were complex, with inverted internal segments of 45-94 kb. In one proband-mother duo, de novo status was inferred by haplotype analysis. Together with two additional patients who harboured smaller predicted protein-truncating variants (p.Arg632^∗^ and p.Ala2223Leufs^∗^13), we estimate the prevalence of this condition in cohorts of patients with unexplained ID to be ~1/1300. Phenotypic blending, present for two cases with additional pathogenic variants in *CASR/PKD1* and *SLC17A5*, hampered the phenotypic delineation of this recently described condition. Our data highlights the benefits of genome sequencing for resolving structural complexity and inferring de novo status. The genomic architecture of 16p13.3 may give rise to relatively high rates of complex rearrangements, adding to the list of loci associated with recurrent genomic disorders.

## 1. Introduction

Over the last 20 years, our understanding of genomic variation has evolved. It is increasingly apparent that repeats and other features present in the underlying genome architecture can act as substrates for the formation of novel structural variants (SVs) [1]. DNA palindromes are one such feature that can form hairpins and cruciform-like secondary structures, which in turn result in a chromosome locus being susceptible to the formation of complex rearrangements. A well-known example is the Xq27.1 palindrome 82 kb centromeric to *SOX3* where intra- and interchromosomal insertions have been reported. Depending on the nature of the integrated sequence, rearrangements involving Xq27.1 can lead to a variety of conditions including hypoparathyroidism [2, 3], hypertrichosis [4] and isolated bilateral vocal cord paralysis [5]. The AT-rich repeat in intron 40 of *NF1* is another example where the palindromic nature of the underlying sequence results in an SV mutational hotspot [6].

A recent meta-analysis of data generated as part of the Deciphering Developmental Disorders (DDD) study (http://www.ddduk.org) and from healthcare settings combined exomes for 31,058 parent-child trios [7]. *SRRM2*, a 15 exon gene that encodes a 2,752 amino acid splicing factor, was one of 28 genes that showed a strong enrichment for de novo mutations, but which had not previously been robustly linked to developmental disorders. Enrichment was driven primarily by protein-truncating variants, with 17/28 of the de novo *SRRM2* variants being frameshift, stop-gain, or disrupting a canonical splice site. *SRRM2* is highly intolerant to putative loss-of-function (pLoF) variants, with 111.4 single nucleotide variants (SNVs) expected vs. 7 observed in gnomAD v2.1.1. It has a LOEUF score = 0.18 and pLI = 1.0, supporting a link to disease via haploinsufficiency [8].

Clinical delineation of this novel syndrome has been undertaken in a cohort of 22 patients with monoallelic *SRRM2* variants [9]. Common findings included developmental/speech delay, autistic traits, attention deficit hyperactivity disorder (ADHD), and overfriendliness. Generalised hypotonia and dysmorphic features were also observed and over half of the cohort was overweight. When present, intellectual disability (ID) was mild.

With evidence of an emerging new syndrome, we searched data from the 100,000 Genomes Project (100kGP) [10] for rare genetic variants likely to result in *SRRM2* haploinsufficiency and six variants were identified in six unrelated probands, hereafter denoted as P1-P6. Prompted by the fact that three of these individuals harboured complex de novo SVs, the nearby genomic architecture was assessed, revealing a palindrome-like structure that we hypothesize results in an increased liability for the formation of such genomic alterations at this locus.

## 2. Editorial Policies and Ethical Considerations

Ethics approval for 100kGP was from Cambridge South REC (14/EE/1112), and participants provided informed consent prior to recruitment.

## 3. Methods

The 100kGP is a UK-wide initiative first conceived in 2012 that aims to provide genetic diagnoses for families with rare disease or cancer and help integrate the use of genome sequencing into the National Health Service [10, 11]. Data from the 100kGP is held in the National Genomic Research Library (doi:10.6084/m9.figshare.4530893.v6), and researchers can apply to access data at http://www.genomicsengland.co.uk/join-a-gecip-domain.

In most rare-disease cases in 100kGP, DNA was extracted from blood and sequencing libraries were prepared using the TruSeq PCR-free high-throughput library preparation kit, followed by 150 bp paired-read sequencing on a HiSeq X machine (Illumina). SVs were called by Manta/Canvas [12, 13] and prioritised using SVRare [14], with an 80% overlap threshold for clustering. In contrast, small variants were identified using Platypus [15] and filtered using the standard clinical tiering pipeline employed by Genomics England [10]. These data were available in the Genomics England research environment using the “tiering_data” table available through the LabKey application. Genomic coordinates for variants are reported on build GRCh38.

## 4. Results

### 4.1. Individuals from 100kGP with SVs Leading to *SRRM2* Haploinsufficiency

**Figure 1:**
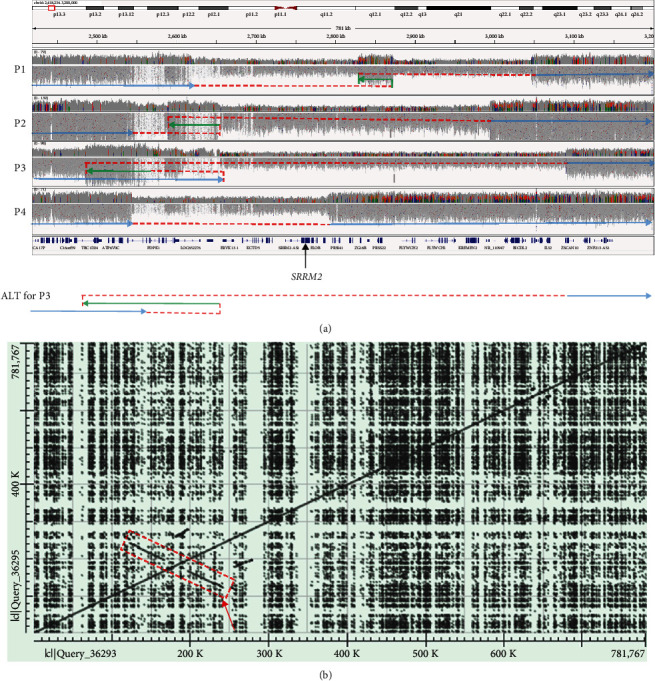
Complex structural rearrangements and genomic architecture at the *SRRM2* locus. (a) IGV read alignments and subway plots showing 4 de novo structural rearrangements leading to *SRRM2* haploinsufficiency. Region shown is 16 : 2,418,234-3,200,000 (GRCh38). Horizontal blue and green arrows indicate noninverted and inverted chromosome segments, respectively, whilst dotted red lines indicate junctions. For Family 3, a second possible configuration that could explain the short-read data is indicated. (b) Dot plot using reference sequence for the same region shown in (a) highlights a palindrome of ~144 kb (red arrow/box). A zoomed-in dot plot is shown in Figure S1.

A review of the SVRare report for *SRRM2* identified four unrelated participants harbouring overlapping SVs that delete the entire coding sequence of *SRRM2* (Figure 1(a)).

The first participant (P1) is a white British male recruited to 100kGP due to ID. Additional features included delayed motor development, delayed speech and language development, and global developmental delay, and he was noted to exhibit aggressive behaviour. Dysmorphic features included a high anterior hairline, hypertelorism, and a broad forehead with parietal bossing. Joint laxity was also present. A 427 kb deletion (16 : 2,619,023-3,046,236) was detected, but a 45 kb segment in the middle (16 : 2,828,415-2,873,007) was retained in an inverted orientation.

P2 is also a white British male recruited to 100kGP due to ID. Additional features included delayed motor development, delayed speech and language development, global developmental delay, autistic traits, and an eye abnormality. A 448 kb deletion (16 : 2,546,884-2,994,539) was detected. Similar to the deletion identified in P1, this deletion contained an internal 61 kb segment (16 : 2,588,852-2,649,945) that had not been deleted but was shown to be in an inverted orientation. This de novo deletion of 16p13.3 had previously been picked up by microarray testing, but at that time, the significance of this finding was unclear.

P3 is a white British female recruited to 100kGP due to ID. Additional features included delayed motor development (sitting at 18 months, standing at 2½, and walking at 4 years), delayed speech and language development, global developmental delay, and microcephaly. The patient had congenital muscular torticollis, bilateral talipes equinovarus, dystonia, and generalised hypotonia. Abnormalities of the eye and gastrointestinal tract were also noted. At age 16 years, she still continues to have low tone and proximal muscle weakness, she is overly social and happy and has significant learning difficulties, functioning as age 5/6 years old. This patient harboured a 482 kb deletion (16 : 2,608,384-3,090,128). Like the variants identified in P1 and P2, this deletion contained an inverted internal segment of 94 kb, but this time, the sequence originated from a nearby region (rather than from within the deleted region) and thus was detected as a duplication (16 : 2,485,590-2,579,759). We note that with short-read data, there are two possible structures that can lead to the pattern of duplication and split-reads (Figure 1(a)). Whilst this ambiguity could potentially be resolved by Bionano optical mapping and possibly long-read sequencing, both configurations result in *SRRM2* haploinsufficiency and so the additional costs would not be justified clinically.

P4 is a white Irish female recruited to 100kGP due to moderate ID. Additional features included hypocalcemia, polycystic kidney disease, delayed motor development and an inability to walk, moderate global developmental delay, and postnatal microcephaly. Previous genetic testing in this individual had uncovered a heterozygous NM_000388.4:c.571G>C; p.(Glu191Gln) variant in *CASR* from her mother and a known pathogenic NM_001009944.3:c.6487C>T; p.(Arg2163Ter) variant in *PKD1* from her father (http://www.ncbi.nlm.nih.gov/clinvar/variation/433972) which explained the hypocalcemia and polycystic kidney disease. Array-based testing had yielded a normal result, albeit at a low resolution. In addition to confirming the previously detected variants, genome sequencing uncovered a 248 kb deletion (16 : 2,543,846-2,792,047). In contrast to the previous 3 cases, no complexity was detected. This individual was therefore suspected to have a blended phenotype linked to variants in three different genes, with the *SRRM2* deletion helping to explain the developmental delay and dysmorphic features that had not been accounted for by the first two genes.

### 4.2. Gene Content and Genomic Architecture on 16p13.3

The minimally deleted region shared by patients P1-P4 includes 5 protein-coding genes (*KCTD5*, *PRSS27*, *SRRM2*, *ELOB*, and *PRSS33*) of which only *SRRM2* has been associated to the disease according to OMIM/PanelApp (https://genome.ucsc.edu/s/AlistairP/SRRM2_100kGP_variants_V2). It should be noted that the deletions in P1 and P3 extend further in the proximal direction and include *THOC6* (MIM #613680, Beaulieu-Boycott-Innes Syndrome) and *CLDN9* (deafness, autosomal recessive 116, provisional, #619093). However, these genes have not been linked to disease via haploinsufficiency as a known disease mechanism and any hemizygous variants in these genes would already have been detected by the clinical tiering pipeline.

For all four SVs, distal breakpoints are localised to a low mapability region (Figure 1(a)) that is annotated in the UCSC genome browser as a segmental duplication. However, analysis of the reference genome using the blast2seq tool (https://blast.ncbi.nlm.nih.gov) highlighted that the region of low mapability also forms part of a ~144 kb palindrome-like structure (Figure 1(b)) that lies approximately 75 kb from *SRRM2*. The approximate coordinates of the palindrome-like structure are chr16 : 2,534,000-2,678,000 (GRCh38), and a zoomed-in dot plot for the region (Figure S1) highlights how the segmental duplications and palindrome are interlinked.

### 4.3. Additional Cases with Small *SRRM2* Variants

Two additional unrelated patients with pLoF *SRRM2* variants were uncovered by searching the tiered SNVs/indels that had been filtered using the standard clinical pipeline employed by Genomics England.

P5 is the first child of unrelated parents. There was antenatal exposure to alcohol, nicotine, and possibly cannabis. She was born by planned Caesarean section at term in view of breech presentation weighing 6 lb and 12 oz. She had an onset of a generalised seizure disorder at 14 months. Brain CT and MRI scan were normal. She sat independently at 10 months and walked at 22 months. Her speech and language were also delayed. She had about 10 single words at age 3 years and started speaking in sentences at age 4½ years. She has her tonsils removed at aged 5 because of recurrent infections. She has had problems with ingrowing toenails. There were no obvious concerns about hearing or eyesight. She had disruptive sleep but this improved with time. She has had chronic constipation requiring 4-6 sachets of Movicol per day, Senna, and diet adjustment. At the assessment age of 9½ years, she was attending a school for children with moderate learning difficulties and required full-time support. Her seizures were controlled with sodium valproate. She was not toilet-trained. She had autistic and ADHD traits. She walked short distances only with her feet turned out and had coordination difficulties. She had a tendency to flap her hands. She enjoyed eating and would eat excessively if allowed to do so. On examination, her head circumference and length were on the 98th centile and her weight on the 99.6th centile. She had a smooth philtrum, full cheeks, fleshy ear lobes, and a high-arched palate but with a normal uvula, slightly tapering fingers, long halluces compared to her other toes, a possible supernumerary nipple on the right side, and a Beighton joint laxity score 2/9 (metacarpophalangeal joints). Some dysmorphic features could be related to antenatal teratogen exposure. Genome sequencing uncovered a 16 bp deletion NM_016333.4:c.6667_6682delGCACCAGCAGCCAACC that predicts a frameshift p.(Ala2223Leufs^∗^13). AACC microhomology was present at either end of this small deletion, which is in contrast to the SVs in P1-4 where no microhomology was identified (Table S1).

The final case (P6) was a white British male where previous genetic analysis had suggested a sialic acid storage disorder due to a maternal NM_012434.5:c.43G>T; p.(Glu15Ter) and paternal c.116G>A; p.(Arg39His) in *SLC17A5*. Although these variants were confirmed by genome sequencing, biochemical testing was not possible as the patient is now deceased. Both variants are listed in ClinVar (http://www.ncbi.nlm.nih.gov/clinvar/variation/167694 and http://www.ncbi.nlm.nih.gov/clinvar/variation/431079) and assessed as pathogenic or likely pathogenic by multiple submitters. The patient had been recruited to 100kGP with a primary diagnosis of epileptic encephalopathy/early onset dystonia; the phenotype was more complex than in the other five cases (P1-5) and included learning disability, behavioural difficulties, ADHD, mild global developmental delay (including delayed walking and speech and language development), atonic drop attacks (which were not felt to be epileptiform in nature), upper limb dystonia, and neonatal hypotonia. Over time, there was evidence of neurodegeneration with an evolving bulbar abnormality causing swallowing difficulties, drooling, and aspiration, in addition to worsening generalised dystonia, increased tone, and deteriorating cognition. He had slow saccadic eye movements but no nystagmus. His downgaze was normal with limited upgaze. There was no evidence of hepatosplenomegaly. Dysmorphic features included a low anterior/posterior hairline, thick hair, high-arched palate, anteverted nares, long palpebral fissures, hypermobile fingers, a short neck, two accessory nipples on the left, inability to protrude his tongue, and severe bilateral plano-valgus deformity. Iron accumulation was identified in both the globus pallidus and the substantia nigra, but was nonprogressive. The patient harboured an NM_016333.4:c.1894C>T; p.(Arg632^∗^) variant in *SRRM2* where the C>T transition is likely due to the deamination of a methylated cytosine at a CpG dinucleotide. The variant was independently returned by DDD for this patient (DECIPHER ID 261425).

### 4.4. De Novo Status and Haplotype Analyses

**Figure 2:**
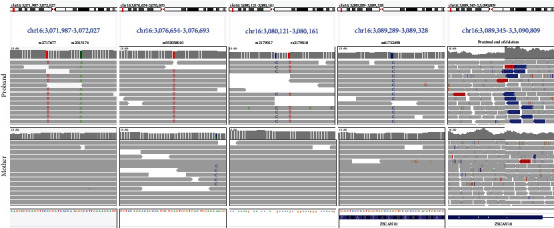
Inference of de novo status of deletion by haplotype analysis. Regions shown are 16 : 3071987-3072027 16 : 3076654-3076693 16 : 3080121-3080161 16 : 3089289-3089328 16 : 3089345-3090809 (GRCh38). Read alignments show 6 SNPs inside the deleted region which are hemizygous for the alternate allele in the proband but homozygous for the reference allele in the mother. The nondeleted chromosome in the proband must have been inherited from the father. The panel on the rights highlights the proximal deletion breakpoint where split read-pairs (blue) and a drop in coverage are seen only in data from the proband. In contrast with the exonic *NEDD4L* deletion (Figure S2a) there was no indication of any mosaicism.

P1-2, P4, and P6 were all recruited to 100kGP as trios and so the respective set of variants described above were confirmed to have arisen de novo due to the absence of the variant in parental genomes, sequenced at similar levels of coverage. P5 was recruited to 100kGP as a singleton, and DNA samples were unavailable for segregation testing; therefore, the inheritance of the heterozygous 16 bp deletion remains unknown. In contrast, P3 was recruited as part of a parent-child duo, with the deletion-inversion not observed in the unaffected mother. Using the genome sequencing data, haplotype analysis uncovered a series of six informative SNPs (rs2717677-rs2015174-rs552058010-rs2179017-rs2179018-rs61732498) within the deleted region, where the nonreference alleles T-A-T-C-T-C were hemizygous in the proband but absent in the mother (Figure 2). The nondeleted chromosome in the patient must have therefore come from the father. From this, it can be inferred that the 482 kb deletion is likely to have arisen de novo.

Haplotype analysis also proved to be useful for P1, where skewed allelic fractions for 30 phased SNVs localised within a de novo deletion involving exons 2-5 of *NEDD4L* (NM_015277.6) suggested that this additional deletion was mosaic in the blood (Figure S2a) and thus of uncertain significance clinically. In tandem with a related read-count analysis (Figure S2b), the genome sequencing data suggested that this deletion was present in 48-64% of cells.

## 5. Discussion

Although array-based genetic testing methodologies are efficient at picking up copy number variants, they are less successful at detecting structural complexity. Whilst whole genome sequencing (ideally with long-reads) is the ideal approach for detecting such events, complex pathogenic SVs remain rare and we note that the pilot study involving the first 2,183 families from the 100kGP did not identify any complex SVs [10]. Disease-focussed studies using data from the 100kGP main programme have been more successful in identifying complex SVs, and examples include a complex inversion in a patient with craniosynostosis [16] and a founder duplication-inversion on chromosome 17 where positional effects on gene expression can result in retinitis pigmentosa [17].

Prompted by the work of Cuinat et al., we searched for pLoF *SRRM2* variants across the 100kGP and identified six unrelated individuals all with ID. Strikingly, 3/6 of our cases harboured de novo complex SVs, each comprising a deletion with an internal inverted segment. In each case, the distal end of the deletion mapped to a 144 kb palindrome-like motif present in GRCh38. In P2 and P3, the de novo deletions of 16p13.3 had previously been identified by microarray testing, but no complexity was recognised. Incomplete characterisation of SVs can lead to misinterpretation of the likely effect on gene function and, furthermore, can impact on the success of genetic validation; for instance, breakpoint PCR would not work in P1-3 described here if the retained/inverted segments are not taken into account when designing primers. We acknowledge that without using long-read sequencing methods, we are unable to rule out the presence of additional sequence complexity at the breakpoints entirely, but given the mechanism of *SRRM2* haploinsufficiency, we consider that this would unlikely alter the clinical interpretation of this set of variants.

Other deletions with breakpoints clustering in this 144 kb palindrome have been reported in the literature [18], and the DECIPHER database (v11.13) includes two such cases (285282 and 306133). If resources allow retesting of these individuals at higher levels of genetic resolution, we suspect that some may also contain additional complexity.

The 1/500 incidence of this condition in the discovery cohort of ID patients described by Cuinat et al. may represent an overestimate of prevalence due to a “winner's curse” type effect better known in the GWAS literature [19]. For the full cohort of 22 subjects [9] assembled with GeneMatcher (https://genematcher.org), the effective denominator is uncertain. A more conservative estimate of prevalence (just including de novo pLoF variants) comes from the Kaplanis et al. study which yields 17/31,058 (i.e., 1/1,827) [7]; however, that is likely an underestimate as exome sequencing does not effectively capture SVs. In this study, P1-5 were all recruited to the 100kGP with ID as the normalised specific disease. Based on the 100kGP data release v15 (26^th^ May 2022), there were 6,517 unrelated individuals recruited under that diagnostic term and so this equates to a prevalence of 5/6,517 (i.e., 1/1,303), a figure which lies in between the two previous estimates. Based on numbers seen for the 100kGP pilot study, around 50% of cases had at least some prior genetic testing done before recruitment (0-16 tests, median 1) [10]. The prevalence of individuals with *SRRM2* loss of function in an ID cohort naïve to prior testing might be expected to be lower.

**Figure 3:**
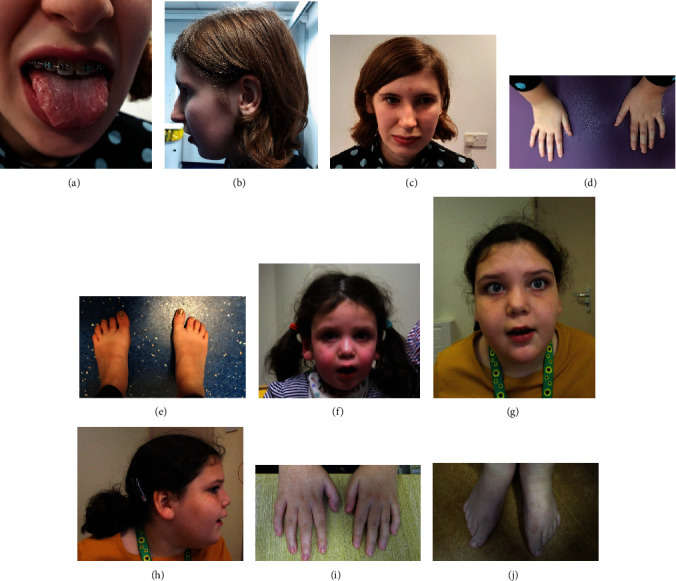
Dysmorphic features seen in P3 (a–e) and P5 (f–j). Clinical photographs showing (a) P3 aged 16 y with geographic tongue, (b) small chin and large ear, (c) thin upper lip and bulbous nose, (d) small hands, and (e) small feet with large halluces. (f) P5 aged 3 y and 3 m with smooth philtrum, thin upper lip, and full cheeks. (g) P5 aged 9 y and 9 m showing smooth philtrum, full cheeks, (h) fleshy ear lobes, (i) tapering fingers, and (j) long halluces compared to other toes.

Our findings help extend the reported gene to phenotype association [9], and a detailed comparison of clinical information for the 6 cases described here with the 22 published cases is shown in Table S1. ID was observed in all 6 individuals described here and ranged from moderate to severe, whereas in the published cohort, the level of ID was typically mild. Microcephaly was reported for half the individuals described here but only in 1/22 of the previous cases. Similarly, seizures were present in 2/6 of our patients but this feature was not reported previously. Over 50% of the published cohort [9] were described to be overweight and that was also noted in two of our patients. Other findings common to both cohorts include autistic/ADHD features and neonatal hypotonia. Dysmorphic features were also seen in both groups of patients, and consented photographs from P3 (Figures 3(a)–3(e)) and P5 (Figures 3(f)–3(j)) are shown. A number of features occur in more than one case, and this includes geographic tongue (Figure 3(a)), large ears/earlobes (Figures 3(b) and 3(h)), thin lips and a bulbous nose (Figures 3(c), 3(f), and 3(g)), small hands with tapering fingers (Figures 3(d) and 3(i)), and large halluces (Figures 3(e) and 3(j)).

Although further clinical comparisons are summarised in Table 1, we note that detailed phenotypic delineation efforts are somewhat hindered by the fact that 2/6 cases (P4 and P6) likely represent blended phenotypes. Dual diagnoses are not uncommon, with early clinical exome studies finding that 4.6-4.9% of molecularly diagnosed cases comprised blended phenotypes resulting from two single-gene defects [20, 21]. Although blended phenotypes involving more than two genes (as seen for P4) have been reported in the literature [22], these are considerably rarer. There could also be a phenotypic consequence of the antenatal exposure to teratogens in P5.

Interestingly, P1 also harboured a second de novo deletion involving *NEDD4L*, a gene linked to periventricular nodular heterotopia [23]. The genome sequencing data allowed us to assess allelic skew and relative read counts and hence demonstrate that this deletion was mosaic in blood, present in around 48-64% of cells. Detectable clonal mosaicism is relatively rare [24] and it is impossible to say what the distribution of this *NEDD4L* deletion is in tissues other than blood. Combined with the lack of MRI data, it is presently unclear the extent to which this additional SV is contributing to the patient's phenotype.

As *SRRM2* encodes an arginine-rich protein (468/2,752 residues), it contains a relatively high number of CpG dinucleotides rendering it a high-risk gene for de novo nonsense variants via deamination of methylated cytosine. Of the 22 variants reported by Cuinat et al., 4 were Arg - > Ter variants. The p.(Arg632^∗^) we report in P6 lies in the first of the two arginine- and serine-rich domains, very close to the previously reported p.(Arg628^∗^) variant and is consistent with that mutational mechanism. However, our study also shows that *SRRM2* is prone to the accumulation of pLoF variants due to a second feature of local genomic architecture. We believe that the complex SV formation in the 3 individuals described here was likely due to a palindrome-like segment that predisposes to the formation of such SVs.

In future, the collection of large cohorts of patients with pLoF mutations in *SRRM2* may facilitate RNAseq studies, which could help determine the downstream consequences of *SRRM2* haploinsufficiency. Such transcriptome studies have been performed on 5 individuals with disruptive variants in *HNRNPR*, a gene which encodes an important component of the spliceosome C complex, indicating that downstream effects included the impairment of homeobox gene expression [25].

In summary, this study highlights the benefits of genome sequencing for resolving SV complexity, detecting mosaicism and for inference of de novo status in parent-child duos. We propose that the 144 kb palindrome on 16p13.3 can lead to high rates of complex SVs at this locus, thus adding *SRRM2* haploinsufficiency to the list of recurrent genomic disorders.

## Figures and Tables

**Table 1:** Comparison of genetic and clinical details between 6 individuals from the 100K Genomes Project with loss of function variants in *SRRM2* and the 22 cases reported previously.

	Cases reported here (*N* = 6)	Cases reported by [9] (*N* = 22)
*SRRM2* variant type	1 frameshift, 1 nonsense, 4 microdeletions (3 complexes)	12 frameshifts, 8 nonsenses, 2 microdeletions
Inheritance	de novo in 5/6†	de novo in 19/22
Prevalence	5/6517 in ID cases from 100kGP (v15 26th May 2022)	2/1000 in the discovery cohort
Gender	3 M, 3 F	14 M, 8 F
Ethnicity	6/6 white	NA
Age at last assessment	5-16 y	4-28 y (mean = 11 y)
Phenotype blending	2/6	NA
Developmental delay	6/6	22/22
Language delay	6/6	16/19
Walking delay	6/6 (1 mild)	8/22
Intellectual disability	6/6 (moderate to severe)	16/20
Seizures	2/6	Not reported
Microcephaly	3/6	1/22 (also 2 with macrocephaly)
Autistic features	4/6	9/22
ADHD features	5/6	6/22
Hypersociability/friendliness	3/6	8/22
Anxiety	3/6	2/22
Hyperphagia	2/6	4/22
Feeding difficulties	4/6	5/22
Neonatal hypotonia	4/6	4/22
Hypotonia at the last assessment	2/6 (also 1 with increased tone)	9/22
Dystonia	2/6	NA
Coordination trouble/dyspraxia	4/6	5/22
Overweight	2/6	12/22
Obesity	1/6	7/22
Tall stature	2/6	4/22
Facial dysmorphism	6/6	20/22
Geographic tongue	2/6‡	NA
Small/short hands and feet	2/5	6/22
Scoliosis	2/6 (1 mild)	1/22 (with hemivertebra)
Planovalgus	2/6	NA
Strabismus	2/6	4/22
Hypermetropia	1/6	3/22
Kidney anomaly	1/3 (L-hydronephrosis and 2 renal cysts)	1/22 (unilateral hypoplastic kidney)
Micropenis/small testes	1/3 (absent testis)	1/14 (both)

†For P3, de novo status is inferred by haplotype analysis (Figure 2). ‡Shown for P3 in Figure 3(a).

## Data Availability

Data from the 100kGP is held in the National Genomic Research Library (doi:10.6084/m9.figshare.4530893.v6), and researchers can apply to access data at http://www.genomicsengland.co.uk/join-a-gecip-domain.

## Supplementary Materials

Supplementary Materials The file “SRRM2_supplementary_figures.docx” contains 2 supplementary figures: “Figure S1: dot plot showing interlinked palindrome and segmental duplications on 16p13.3” and “Figure S2: genome sequencing data indicates that an exonic deletion involving *NEDD4L* is mosaic”. In addition, “Table S1.pdf” contains “Table S1: Genetic and clinical details for 6 individuals from the 100K Genomes Project with loss of function variants in *SRRM2*”.
