# Supplementary material for: A Palindrome-Like Structure on 16p13.3 Is Associated with the Formation of Complex Structural Variations and SRRM2 Haploinsufficiency
Source: Hum Mutat. 2023 Apr 11;2023:6633248. doi: 10.1155/2023/6633248 (PMC11918891; doi:10.1155/2023/6633248)
Supplement: Supplementary Materials — The file “SRRM2_supplementary_figures.docx” contains 2 supplementary figures: “Figure S1: dot plot showing interlinked palindrome and segmental duplications on 16p13.3” and “Figure S2: genome sequencing data indicates that an exonic deletion involving NEDD4L is mosaic”. In addition, “Table S1.pdf” contains “Table S1: Genetic and clinical details for 6 individuals from the 100K Genomes Project with loss of function variants in SRRM2”. [file 6633248.f1.zip › SRRM2_supplementary_figures.docx]

***BRIEF REPORT***

**A palindrome-like structure on 16p13.3 is associated with the formation of complex structural variations and *SRRM2* haploinsufficiency**

***Supplementary Figures***


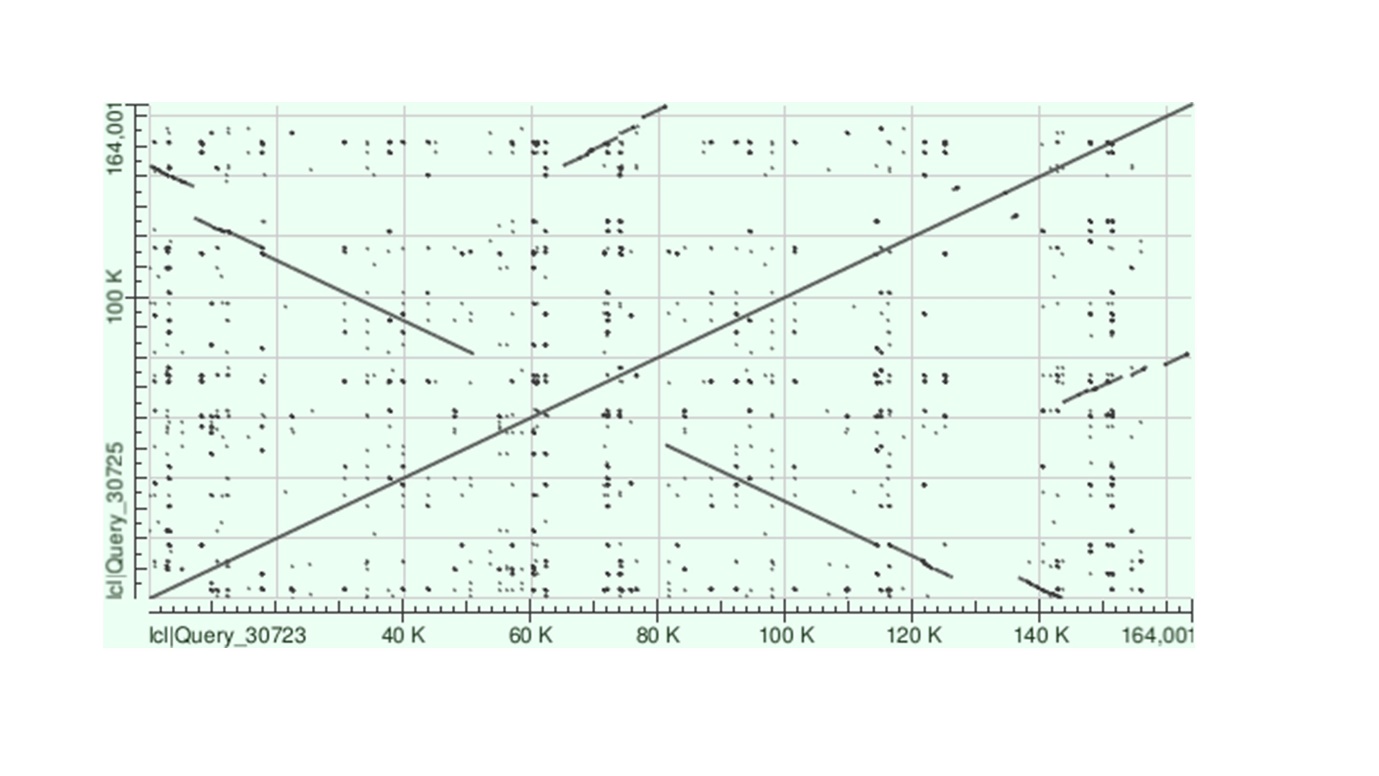


**Figure S1:** Dot plot showing interlinked palindrome and segmental duplications on 16p13.3. Analysis is based on 164kb of genomic sequence from chr16:2534000-2698000 of the GRCh38 reference genome. The palindromic component of this complex structure corresponds to chr16:2534000-2678000.


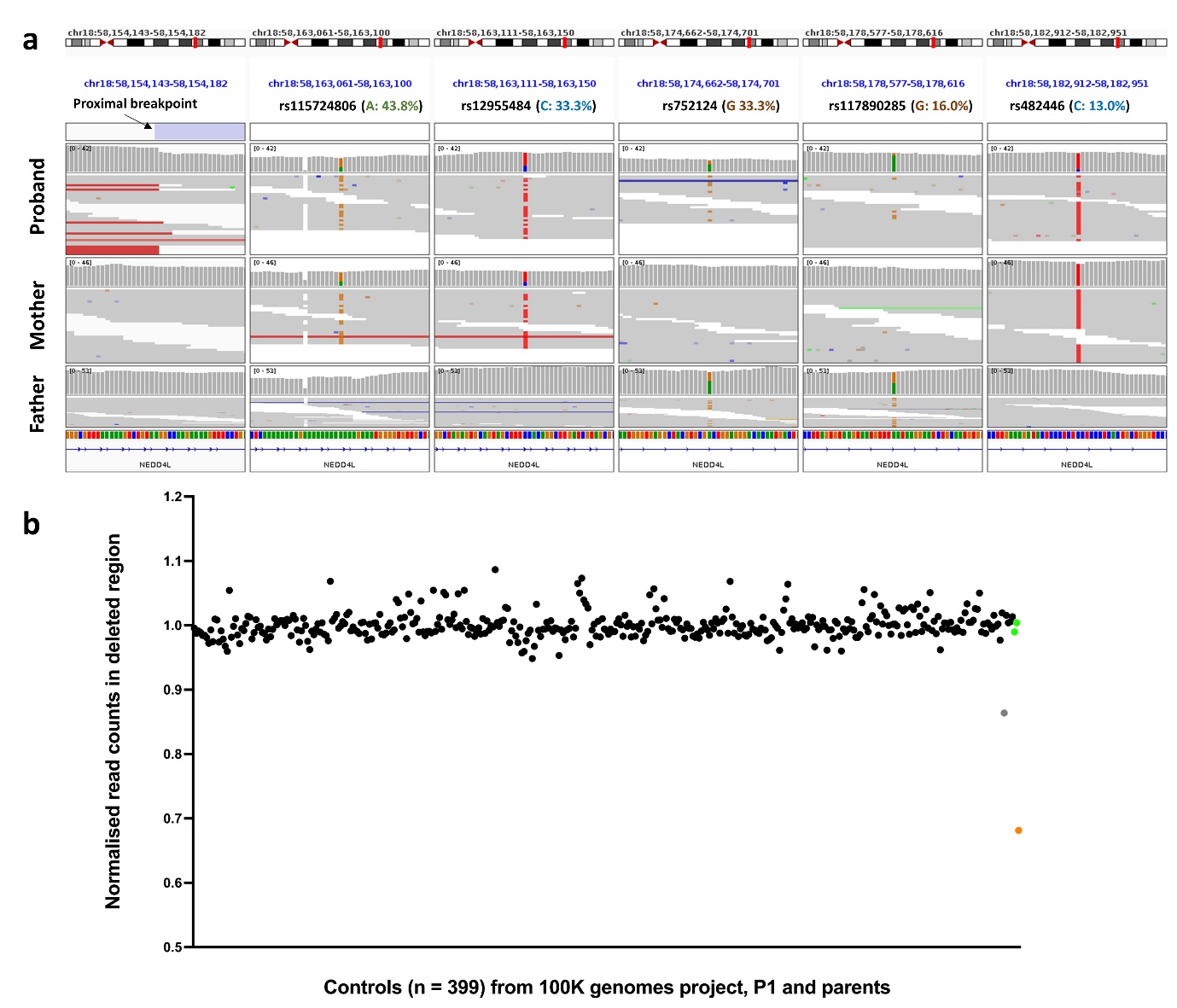


**Figure S2:** Genome sequencing data indicates that an exonic deletion involving *NEDD4L* is mosaic. a) Retained heterozygosity within the 144kb deletion is indicative of clonal mosaicism. In P1, 31 high confidence “heterozygous” SNVs were identified within the deleted region, of which 30 could be phased by inheritance. Alignments supporting a representative set of five SNVs closest to the proximal end of the deletion are shown in IGV using the “squished” option. Consistent allelic skewing shows the paternal allele (labelled in parentheses) is always be the minor allele. The mean paternal allelic fraction across this set of SNVs allowed us to estimate the deletion to be present in approximately 48% of cells. Coordinates are based on build GRCh38. b) In a related analysis, samtools was used to extract read count information from the deleted region and this data was normalised to the number of reads present elsewhere on chromosome 18. These ratios were then compared to the mean ratio from 399 control samples sequenced as part of the same delivery date batch when P1 was sequenced. Whilst the parents (green) showed normal read counts, the ratio seen in proband (red) was 68%, which translates to the deletion being present in ~64% of blood cells. Except for single outlier (grey), the controls all cluster around 100%.
