# Supplementary material for: A Palindrome-Like Structure on 16p13.3 Is Associated with the Formation of Complex Structural Variations and SRRM2 Haploinsufficiency
Source: Hum Mutat. 2023 Apr 11;2023:6633248. doi: 10.1155/2023/6633248 (PMC11918891; doi:10.1155/2023/6633248)
Supplement: Supplementary Materials — The file “SRRM2_supplementary_figures.docx” contains 2 supplementary figures: “Figure S1: dot plot showing interlinked palindrome and segmental duplications on 16p13.3” and “Figure S2: genome sequencing data indicates that an exonic deletion involving NEDD4L is mosaic”. In addition, “Table S1.pdf” contains “Table S1: Genetic and clinical details for 6 individuals from the 100K Genomes Project with loss of function variants in SRRM2”. [file 6633248.f1.zip › Table S1_revision.pdf]

Table S1: Genetic and clinical details for 6 individuals from the 100K Genomes Project with loss of function variants in SRRM2. †, indicates position is estimated by Canvas as breakpoints in repetitive region. NA, not known or not applicable.

Family ID	P1	P2	P3	P4	P5	P6	Summary from Cuinat *et al* 2022 (N=22, unless indicated otherwise)	Comments and summary of present cohort
***SRRM2* variant type**	Deletion with internal segment retained/inverted	Deletion with internal segment retained/inverted	Deletion with nearby duplicated sequence inserted in inverted orientation	Microdeletion	Frameshift	Stop-gain	12 frameshift, 8 nonsense, 2 microdeletions	Rearrangement in F3 is ambiguous with short reads but does not affect clinical interpretation
**Variant coordinates: GRCh38 (SVs) or NM_016333.4 (SNV/indels)**	427kb deletion (16:2,619,023-3,046,236) with a 45kb segment in the middle (16:2,828,415-2,873,007) not deleted but inverted	448kb deletion (16:2,546,884†-2,994,539) with a 61kb segment in the middle (16:2,588,852-2,649,945†) not deleted but inverted	482kb deletion (16:2,608,384-3,090,128) with an inverted 94kb duplication (16:2,485,590-2,579,759) in the middle	248kb deletion (16:2,543,846-2,792,047)	Heterozygous c.6667_6682delGCACCAGCAGCCAACC; p.(Ala2223Leufs*13)	c.1894C>T; p.(Arg632*) which is C>T at CpG dinucleotide	See fig. 1A in Cuinat *et al* 2022	All 4 SVs have at least one breakpoint that lies in a ~120kb palindrome on 16p13.3 (Figure 1). DECIPHER cases 285282 and 306133 with *de novo* deletions of this locus also have breakpoints in the same palindrome so likely genomic architecture predisposes to rearrangements. Similar to locus downstream of *SOX3* (PMID: 35095096). Without WGS data then complexity may not be recognised and so might make validation e.g. by PCR-Sanger very difficult.
**Breakpoint information**	No microhomology detected. One of the internal breakpoints is in MER58B repeat.	No microhomolgy detected but distal end of inversion in Charlie1 repeat.	No microhomology but distal end of deletion in AluSx, distal end of inversion in MER21A, proximal end of inversion in AluSz6	No microhomology but proximal end is at polyA stretch (14xA) and both ends involve AluSx elements	AACC microhomology	N/A	N/A	Microhomology detected in 1/6 and Alu elements in 2/6
**Inheritance (zygosity)**	*de novo* (het)	*de novo* (het)	*de novo* (het) - inferred from haplotype; rs2717677-rs2015174-rs552058010-rs2179017-rs2179018-rs61732498; T-A-T-C-T-C not seen in mother	*de novo* (het)	Unknown - parental samples unavailable (het)	*de novo* (het)	*de novo* in 19/22	*de novo* in 5/6. F3 is just mother-child duo and nice example of inferring *de novo* status without paternal sample (Figure 2)

**Ascertainment**	Genome sequencing by Genomics England and Manta/Canvas calls analysed with SVRare				Genome sequencing by Genomics England and Platypus variant calls analysed with clinical TIERING pipeline		First two cases from cohort of developmental delay and intellectual disability from Nantes hospital and clinical exome sequencing. Other cases from GeneMatcher	2/1000 in initial cohort from Cuinat *et al* likely an overestimate (winner's curse?). Hard to say what denominator is from GeneMatcher. Prevalence of cases in 100kGP recruited under Intellectual disability is 5/6,517 [data release v15 26 May 2022; ignore F6 as was recruited under different diagnostic category]
**Prior testing - array (resolution, result)**	arr[GRCh37] 16p13.3(2747962_2869281)x1,16p13.3(2941230_3076495)x1 *de novo* but of uncertain significance. Agilent design 028469 with median resolution of 120kb	*de novo* 16p13.3 deletion of uncertain significance	16p13.3 microdeletion - uncertain significance	60K array - normal. Array testing in other family members picked up a different rare deletion on 16p13.3 of 297kb (16:6,655,575-6,952,079) and a 839kb tandem-duplication on 3q22.1 (3:132,365,682-133,204,467) in affected brother, unaffected mother and grandmother - these are of uncertain significance and were not transmitted to the proband.	array CGH - normal	array CGH - normal	CytoSNP-850K (Illumina) for patients 15 and 22	*The de novo* deletions seen in P1-3 were all picked up by arrays prior to WGS but at last review they were VUS (Kaplanis *et al* was published in Oct 2020). Only for P1 was complexity detected. For P4, whom has the smallest deletion, it was missed by original array testing at 60K resolution
**Prior testing - exome**	No	No	NA	NA	NA	Yes (DDD)	NA	1/6 but limited information
**Prior testing - other (single gene/panel NGS/karyotyping/biochem etc)**	None	No	NA	Testing of *CASR* and *PKD1* (see below)	NA	*WDR45* and *C19orf45* (research lab). Patient had normal karyotype, creatine kinase, alpha-fetoprotein, immunoglobulins, mucopolysaccharide screen, urate cholesterol studies, urine purine screen, very long chain fatty acids, transferrin isoforms, lysosomal enzymes (white cell and plasma screen), copper, ceruloplasmin, and amino acids with urinary amino and organic acids. Biochemistry for Salla	NA	2/6 had targeted sequencing but limited information

						disease (Neu5Ac) was not performed.		
**Clinical details**								
**Main diagnosis**	Intellectual disability	Intellectual disability	Intellectual disability	Intellectual disability	Intellectual disability	Epileptic encephalopathy/early onset dystonia	Intellectual disability and developmental delay	ID in 5/6
**Gender**	M	M	F	F	F	M	14M, 8F	3M, 3F
**Ethnicity**	White British	White British	White British	White Irish	White British	White British	NA	6/6 white
**Age at last assessment**	6 years	5y 2m	16 years	10y 6m	9y 9m	NA (now deceased)	4-28y (mean = 11y)	5-16y
**Phenotype blending and details of other variants**	*de novo* 142kb deletion of *NEDD4L* involving exons 2-5 (NM_015277.6). GRCh38 coordinates are 18:58154163-58296575. Mosaic in blood DNA so of uncertain significance and no MRI testing available to assess whether PVNH is present. Mosaicism in 48-64% of cells depending on how it is calculated (Fig. S2).	NA	NA	*CASR*-related AD hypocalcemia from mother due to NM_000388.4:c.571G>C; p.(Glu191Gln) and AD PKD1 from father due to NM_001009944.3: c.6487C>T; p.(Arg2163*) www.ncbi.nlm.nih.gov/clinvar/variation/433972	Missense variant in *GABBR2* NM_005458.8:c.137C>G p.(Ala46Gly) (PM2, PP2, BP4 - warm class 3 variant) - VUS in ClinVar www.ncbi.nlm.nih.gov/clinvar/variation/850847	Sialic acid storage disorder due to maternal NM_012434.5:c.43G>T; p.(Glu15Ter) and paternal c.116G>A p.(Arg39His) in *SLC17A5* - both in ClinVar www.ncbi.nlm.nih.gov/clinvar/variation/167694 and www.ncbi.nlm.nih.gov/clinvar/variation/431079. Biochemical tests all normal.	NA	2/6 are likely blended phenotype and 2 others where potential contributory variation of uncertain significance. Dual diagnoses relatively common but less so with 3 variants (see PMID:26813946)
**Neurodevelopment and behaviour**								
**Developmental delay**	Yes	Yes	Yes	Yes	Yes	Yes	22	6/6
**Language delay**	Yes	Yes	Yes	Yes	Yes, 10 words at age 3y	Yes -mild	16/19	6/6

**Walking delay**	Yes	Yes	Yes - 4 years	Yes	Yes, 22 months	Yes - mild	8	6/6 (1 mild)
**Intellectual disability**	Yes	Yes	Yes - currently function at 5/6 year level at 16 years	Yes (moderate to severe LD)	Yes - school for moderate learning difficulties	Yes - severe	16/20	6/6 (moderate to severe)
**Seizures**	No	No	No	Hypocalcemic seizures on D5, Febrile seizure @2 yrs (normal EEG)	Generalised seizures, onset at 14 months, controlled with sodium valproate	No, atonic drop attacks felt not to be epileptiform	not reported	Seizures not mentioned in Cuinat *et al.* 2/6 in present cohort
**Microcephaly**	No	Yes	Yes	Yes (postnatal)	No	No	1 (and 2 with macrocephaly)	Cuinat *et al* only mention in main text. 3/6 in present cohort
**Autistic features**	No	Yes	Yes - mild	No	Yes	Yes	9	4/6
**ADHD features**	No	Yes	Yes	Yes	Yes	Yes	6	5/6
**Hypersociability /friendliness**	No	No	Yes	Yes	Yes	No	8	3/6
**Anxiety**	No	No	Yes	No	Yes	Yes	2	3/6
**Hyperphagia**	No	No	Yes	No	Yes	No	4	2/6
**Feeding difficulties**	No	Vomiting, dairy allergy	As a baby yes, now improved	No	Early on	Reduced oral intake	5	4/6
**Other neurological findings**								
**Neonatal hypotonia**	No	No	Yes	Yes	Yes	Yes	4	4/6
**Hypotonia at the time of last assessment**	No	No	Yes, low tone	Yes, low tone	No	No, hypertonia	9	2/6 (and 1/6 with increased tone)
**Dystonia**	No	No	Yes - torticollis	No	No	Yes (predominantly upper limbs)	NA	2/6
**Distal hyperlaxity**	No	No	No	No	No	No	4	0/6
**Coordination trouble/dyspraxia**	No	No	Yes	Yes	Yes	Yes but likely secondary to dystonia	5	4/6
**Growth**								
**Overweight**	Yes	No	No - but only because parents control diet	No	Yes	No (swallowing difficulties and a tendency to undereat)	12	2/6
**Obesity**	No	No	No	No	Yes	No	7	1/6
**Tall stature**	No	No	Yes	No	Yes	No	4	2/6
**Morphological features**								
**Facial dysmorphism**	Yes (wide forehead, prominent parietal bossing, high frontal hairline, mild hypertelorism, long eyelashes)	Arched brows, narrow palpebral fissures, small, crumpled ears	Yes (small chin, large, thin upper lip and bulbous nose)	Yes (prominent forehead, short nose, long and smoothish philtrum with thin upper lip, mild hypertelorism)	Yes (smooth philtrum, full cheeks, fleshy ear lobes, high arched palate and thin upper lip - but the latter is similar to maternal grandmother's)	Yes	20	2/6 with hypertelorism, 2/6 with smooth philtrum, 2/6 with prominent/wide forehead (Fig), 2/6 with thin upper lip vs 7/22

**Small/short hands and feet**	No	Short 5th fingers	Yes	NA	No	No	6	2/5
**Ophthalmologic abnormalities**								
**Strabismus**	No	No	Yes	Yes	No	No	4	2/6
**Hypermetropia**	No	No	No	Yes	No	No	3	1/6
**Other visceral and skeletal abnormalities**								
**Kidney anomaly**	NA	No	NA	Yes (L-hydronephrosis, 2 renal cysts)	NA	No	1 (unilateral hypoplastic kidney)	1/3
**Complex congenital heart defect**	No	No	No	No	No	No	1	0/6
**Micropenis, small testes**	No	Penis normal, 1 testis absent	NA	NA	NA	No	1/14 (males)	1/3 (absent testis)
**Spina bifida**	No	No	No	No	NA	No	1	0/5
**Scoliosis**	No	No	Yes (mild)	Yes (thoracic)	No	No	1 (with hemivertebra)	2/6
**Other information**								
**Validation/ reporting status**	NA - already picked up by array prior to WGS	Validated in NHS lab December 2016	NA - already picked up by array prior to WGS	Repeat array has confirmed the *SRRM2* deletion (227kb)	Sample taken to validate (Oct 2022)	Biallelic variants in *SLC17A5* reported by 100KGP. *SRRM2* variant reported initially by DDD.	-	5/6 (1 is awaited)

**Other findings**	He has an increasingly challenging and aggressive behaviour - at the point he will not go to school with the minibus as he would attack the chaperone and driver. He is now overweight. He has frequent tonsillitis and ear infections. Suffers from glue ears. Hearing check was normal.	Sensory behaviour and toe walking. Complains of leg pain of unknown cause. Thin skin and visible veins with eczema. Geographic tongue. Staring episodes but no evidence that these are ictal.	Now 16y and has improved as got older, but learning still significantly limited at an age 5/6 level. She is very happy though and smiley. She has improved with her tone but has planovalgus feet and wears insoles, she has leg length discrepance and had 8 plates on the leftside. Her lower limb proximal muscles are weaker 4-4+/5 MRC distally strong. She has a geographical tongue. She has only one lens in her eyes. Renal USS are being arranged as she reports frequent UTIs as a young child - and as there have been no scans to date, the presence of kidney abnormalities is uncertain. Normal brain and spinal MRIs.	Now 10½ years old and in special needs school. Speech and tone have improved. Recent diagnosis of clinically obvious thoracic scoliosis. Happy, friendly and impulsive child with outbursts of aggression. Current weight 30kg, height 130cm, HC 46.5cm.	Disruptive sleep early on - improved by age 9y; severe chronic constipation; in-growing toe nails; slightly tapering fingers; long halluces compared to other toes; tendency to hand flap; possible supernumerary nipple on the right side. Antenatal exposure to teratogens	Progressive neurodegeneration, iron deposits in the brain and a thin corpus callosum, involuntary movements, spasticity of the left leg, bilateral planovalgus, swallowing difficulties and dysarthria (likely bulbar palsy), limited tongue movements, long slender fingers with lateral deviation of the first three fingers, slow upgaze.	-	2/6 have geographic tongue (Fig 3a); 2/6 with planovalgus (often goes with the hypotonia)
